# Supplementary material for: Optimized superconductivity in the vicinity of a nematic quantum critical point in the kagome superconductor Cs(V1-xTix)3Sb5
Source: Nat Commun. 2023 Jul 6;14:3899. doi: 10.1038/s41467-023-39495-1 (PMC10326258; doi:10.1038/s41467-023-39495-1)
Supplement: Supplementary file 1 — Supplementary Information [file 41467_2023_39495_MOESM1_ESM.pdf]

Supplementary information for "Optimized superconductivity in the vicinity of a nematic quantum critical point in the kagome superconductor $Cs(V_{1-x}Ti_x)_3Sb_5$"

Yeahan Sur,[1] Kwang-Tak Kim,[1] Sukho Kim,[1] and Kee Hoon Kim[1, 2*]

[1] *CeNSCMR, Department of Physics and Astronomy, Seoul National University, Seoul 08826, Republic of Korea*

[2] *Institute of Applied Physics, Department of Physics and Astronomy, Seoul National University, Seoul 08826, Republic of Korea*

*E-mail: optopia@snu.ac.kr

Supplementary Note 1. The residual resistivity (ρ_0) and the residual resistivity ratio (RRR) of CsV_3Sb_5

After the discovery of CsV_3Sb_5 in 2019[1], single crystals of CsV_3Sb_5 have been grown by numerous groups using the Cs-Sb flux method[2–4]. However, it has been found that the sample qualities of the single crystals differ greatly from group to group, with the residual resistivity ratios (RRR) varying from RRR = 16–118[2–4]. Moreover, the reported charge density wave transition temperatures T_{CDW}'s have also exhibited a variation between the range of T_{CDW} = 92.2–95.4 K[2–4]. Supplementary Table 1 shows a comparison of the residual resistivity (ρ_0), RRR, and T_{CDW} of CsV_3Sb_5 from values obtained from literature. It can be seen that the residual resistivity ρ_0 from this work exhibits the lowest value of 0.65 μΩ cm, indicating the lowest amount of impurity scattering as compared to single crystals reported in literature. Moreover, the RRR from this work exhibits the highest value of 129.2, suggesting a high quality of the CsV_3Sb_5 single crystal studied in this work. Interestingly, it is noted that the single crystals with low RRR values tend to exhibit lower a value of T_{CDW}. For example, T_{CDW} = 92.2 K is found in a single crystal with an RRR of 16[4], while T_{CDW} = 95.4 K is found in a single crystal with an RRR of 118[2]. In this work, a T_{CDW} = 98 K is found in a single crystal with an RRR = 129.2, which can be understood by the high quality of the single crystal of CsV_3Sb_5 studied in this work.

Supplementary Note 2. Capillary XRD and EPMA measurements of $Cs(V_{1-x}Ti_x)_3Sb_5$ single crystals.

To investigate the relationship between doping and lattice constants, X-ray diffraction patters of $Cs(V_{1-x}Ti_x)_3Sb_5$ single crystals have been measured using the capillary method. An XRD pattern for ground single crystals of $Cs(V_{0.98}Ti_{0.02})_3Sb_5$ (black dots) and the Rietveld refinement result (red line) from the FullProf software are shown in Supplementary Figure 1**a**. The refinement considering a preferential orientation along the *c*-axis could well reproduce the XRD pattern, resulting in R_{wp} = 28.1 and χ^2 = 1.67. Similar analyses has been done in the other doping values as well with R_{wp} values less than 30 and χ^2 values less than 2. As a result, the refined *a* and *c* values have been obtained as in Supplementary Figure 1**b**–**c**. It can be seen that both *a* and *c* parameters exhibit a decreasing trend with increased doping ratio. This effect can be understood by the smaller ionic radii of Ti^{4+} (60.5 pm) as compared to the V^{3+} (64 pm) and supports that Ti atoms enter the V site.

In order to quantitatively measure the actual V: Ti ratio in $Cs(V_{1-x}Ti_x)_3Sb_5$, wavelength dispersive x-ray spectroscopy (WDS) were performed. Supplementary Figure 2**a** depicts a scanning electron microscope (SEM) image of a $Cs_{1.01}V_{2.919}Ti_{0.081}Sb_5$ single crystal. One can immediately find that the atomic ratio of V ~ 2.919 is lower than 3, while the sum of the atomic ratios of Ti and V approximately equals to a value of ~ 3. This indicates that the Ti atoms replace the V atoms upon doping, which is in-line with the evolution of the lattice constants obtained from the XRD refinements. Supplementary Figure 2**b** shows the plot of nominal V: Ti ratio ($x_{nominal}$) vs. actual V: Ti ratio (x_{WDS}) as obtained from the WDS analysis. It could be seen that the actual V: Ti ratio closely follows the nominal V: Ti ratio at least up to $x_{nominal}$ = 0.05. For $x_{nominal}$ = 0.06, the x_{WDS} exhibits a slight upward deviation from the linear guide line indicating a higher doping ratio as compared to the nominal value.

Supplementary Note 3. Comparison of the phase diagram

Supplementary Figure 3 shows the phase diagram of $Cs(V_{1-x}Ti_x)_3Sb_5$; T_{CDW} (orange circles) is obtained from the data of ρ_{ab} and T_c (orange triangles) is obtained from the criterion of $0.5\rho_N$ (ρ_N: normal-state resistivity). To compare the phase diagram of this work with other phase diagrams of $Cs(V_{1-x}Ti_x)_3Sb_5$ reported in literature, data obtained from two different groups are overlaid on top of Supplementary Figure 3; T_{CDW} and $T_c \times 5$ from ref. 5 is shown in blue octagons and blue diamonds, while $T_c \times 5$ from ref. 6 is shown in violet squares. The T_{CDW} from ref. 6 is not shown since the lowest Ti doped sample $Cs(V_{0.949}Ti_{0.051})_3Sb_5$ in ref. 6 does not exhibit a CDW transition. It could be immediately seen that the evolution of T_c with Ti doping ratio x exhibits comparable values between this work and refs. 5-6. This indicates the reproducibility of T_c between these independent phase diagrams of $Cs(V_{1-x}Ti_x)_3Sb_5$. However, the T_{CDW} in this work exhibits higher T_{CDW} values as compared to ref. 5, indicating a discrepancy of the T_{CDW} between two works.

In order to understand the discrepancy of T_{CDW} between this work and ref. 5, temperature dependence of the in-plane resistivity ρ_{ab} normalized to the value at 300 K ($\rho_{ab}/\rho_{ab,300\,K}$) in $Cs(V_{1-x}Ti_x)_3Sb_5$ is plotted in Supplementary Figure 4**a**–**d**. As the $Cs(V_{1-x}Ti_x)_3Sb_5$ single crystals with identical doping ratios are available for four doping ratios of $x = 0$, 0.01, 0.03, 0.05, the $\rho_{ab}/\rho_{ab,300\,K}$ between the doped single crystals of this work and ref. 5 can directly compared with each other. It is noted that the RRR values of this work is higher than the values of ref. 5 for all single crystals with $0 \leq x \leq 0.05$. Considering that single crystals of CsV_3Sb_5 with higher RRR exhibits a higher T_{CDW} (Supplementary Table 1), the higher T_{CDW} in this work may be associated with the higher RRR values of each sample as compared to ref. 5.

Supplementary Note 4. Theoretical background for the nematic susceptibility in the D_{6h} symmetry group

According to the general definition of elastoresistance coefficients[7]

$$(\Delta R/R)_i \equiv \sum_{j=1}^{6} m_{ij}\varepsilon_j, \tag{1}$$

$\partial N/\partial(\varepsilon_{xx}-\varepsilon_{yy})$ can be expressed in terms of m_{ij}, where ε_j represents the engineering strain, the m_{ij} are elastoresistance tensor components, and the subscripts *i* and *j* represent the Voigt notation (1 = *xx*, 2 = *yy*, 3 = *zz*, 4 = *yz*, 5 = *zx*, 6 = *xy*).

Particularly, for a crystal in the D_{6h} point group with *x* along the [100] axis, the elastoresistance tensor m_{ij} in the absence of magnetic field can be expressed as[8]

$$m_{ij}^{D6h} = \begin{pmatrix} m_{11} & m_{12} & m_{13} & 0 & 0 & 0 \\ m_{12} & m_{11} & m_{13} & 0 & 0 & 0 \\ m_{13} & m_{13} & m_{33} & 0 & 0 & 0 \\ 0 & 0 & 0 & m_{44} & 0 & 0 \\ 0 & 0 & 0 & 0 & m_{44} & 0 \\ 0 & 0 & 0 & 0 & 0 & (m_{11}-m_{12})/2 \end{pmatrix}.$$

By substituting m_{ij}^{D6h} into the definition of the elastoresistance coefficient $\left(\frac{\Delta R}{R}\right)_i \equiv \sum_{j=1}^{6} m_{ij}\varepsilon_j$, one can obtain the following two equations,

$$\left(\frac{\Delta R}{R}\right)_{xx} = m_{11}\varepsilon_{xx} + m_{12}\varepsilon_{yy} + m_{13}\varepsilon_{zz}$$
$$\left(\frac{\Delta R}{R}\right)_{yy} = m_{12}\varepsilon_{xx} + m_{11}\varepsilon_{yy} + m_{13}\varepsilon_{zz}.$$

The subtraction of these two equations results in the following expression

$$m_{11} - m_{12} = \frac{\left(\frac{\Delta R}{R}\right)_{xx} - \left(\frac{\Delta R}{R}\right)_{yy}}{(\varepsilon_{xx}-\varepsilon_{yy})},$$

which becomes $\frac{\partial N}{\partial(\varepsilon_{xx}-\varepsilon_{yy})}$ in the small strain limit of $(\varepsilon_{xx}-\varepsilon_{yy}) \to 0$.

Moreover, according to the irreducible representations of the D_{6h} point group, any arbitrary strain in the hexagonal lattice can be divided into the three strain components[8], $\varepsilon_{A1g} = (\frac{1}{2}(\varepsilon_{xx}+\varepsilon_{yy}),\ \varepsilon_{zz})$, $\varepsilon_{E1g} = (\varepsilon_{xz}, \varepsilon_{yz})$, and $\varepsilon_{E2g} = (\frac{1}{2}(\varepsilon_{xx}-\varepsilon_{yy}), \varepsilon_{xy})$. As the elastoresistance $(m_{11}-m_{12})$ represents the nematic susceptibility induced by $(\varepsilon_{xx}-\varepsilon_{yy})$ strain, $(m_{11}-m_{12})$ represents the nematic susceptibility along the E_{2g} symmetry channel. In conclusion, the elastoresistance coefficient $(m_{11}-m_{12})$ which represents the nematic susceptibility along the E_{2g} symmetry channel can be obtained by measuring the strain-dependent resistivity anisotropy along two perpendicular crystallographic directions, with one axis along the [100] crystal axis.

Supplementary Note 5. Analysis of the temperature-dependent nematic susceptibility above T_{CDW}

Supplementary Figure 5**a** shows the temperature dependence of the nematic susceptibility $\tilde{n}$ for $Cs(V_{0.99}Ti_{0.01})_3Sb_5$. One can find that a jump in $\tilde{n}$ occurs near the T_{CDW}, indicating enhanced elastoresistance anisotropy associated with the formation of the CDW order. Below this jump, $\tilde{n}$ can be well fitted to the Curie–Weiss-type temperature dependence (Eq. (1) in the main text) down to 12 K (top panel of Supplementary Figure 5**a**) with the fitting parameters of C = 157.4 K, θ_{nem} = -4.1 K, and $\tilde{n}_0$ = 13.77 (red solid line). This indicates that Curie–Weiss-type nematic correlations exists above 12 K and at least up to T_{CDW}. In order to understand the nematic correlation effects above T_{CDW}, a magnified figure of the temperature dependence of $\tilde{n}$ is shown in Supplementary Figure 5**b**. Surprisingly, the data above T_{CDW} can be well fitted with the same fitting parameters of C = 157.4 K and θ_{nem} = -4.1 K, only with a different choice of $\tilde{n}_0$ (orange solid line). This indicates that, if the jump of $\tilde{n}_0$ due to the lowered lattice symmetry is considered, the nematic correlation seems to persist even above T_{CDW} for $Cs(V_{0.99}Ti_{0.01})_3Sb_5$. Similar to $Cs(V_{0.99}Ti_{0.01})_3Sb_5$, our measurements reveal that all Ti doped samples $Cs(V_{1-x}Ti_x)_3Sb_5$ with $0 \leq x \leq 0.03$ can be well fitted to the Curie–Weiss-type temperature dependence above T_{CDW} (top panels of Supplementary Figure 6**a**–**h**). For $x \geq 0.04$, Eq. (1) cannot be fitted very well to the $\tilde{n}(T)$ curves due to almost temperature-independent behaviour below and above the T_{CDW} (Supplementary Figure 6**i**–**k**). The parameters obtained from analysis of $\tilde{n}$ are summarized in Supplementary Table 2.

Supplementary Note 6. Comparison between the nematic susceptibility data obtained by the four-probe method and the modified Montgomery method.

The four-probe measurement using two samples has shown to be technically solid[7] and thus has been widely utilized by various groups within recent years[8-11]. However,

measurements performed in two samples can in principle result in realistic errors arising from the difference in strain transmission through the glue layer or from the anisotropy of strain transmission. To double-check the reproducibility of the elastoresistance measurements that measures the E_{2g} symmetry channel, an additional measurement using only one sample[12] has been performed in $Cs(V_{0.9925}Ti_{0.0075})_3Sb_5$, which is close to the nematic quantum critical point of x = 0.009-0.01.

In this case, one $Cs(V_{0.9925}Ti_{0.0075})_3Sb_5$ single crystal was shaped in a rectangular geometry with the sample size of ~ 0.9 mm (L_x) × 0.92 mm (L_y) × 20 μm (L_z). Gold wires of 25 μm were attached to the sample in the modified Montgomery geometry[12] with silver paint (Dupont 4929N) as shown in Supplementary Figure 7**a**. Supplementary Figure 7**b** shows a schematic figure showing the measurement configurations. Resistance along the x-direction (R_{xx}) is measured by the voltage drop across the contacts C and D, while current is sourced across contacts A and B. Similarly, the resistance along the y-direction (R_{yy}) is measured by the voltage drop between B and D, while current is sourced across A and C. It is well known that in the thin sample limit of $L_z/(L_x \times L_y)^{0.5} < 0.5$, the resistivity along the x and y directions can be calculated by the relations [12-13]:

$$\rho_{xx} = \frac{\pi}{8\alpha}\left(\frac{L_y L_z}{L_x}\right) R_{xx}\sinh(\pi\alpha),\ \rho_{yy} = \frac{\pi\alpha}{8}\left(\frac{L_x L_z}{L_y}\right) R_{yy}\sinh(\pi/\alpha),$$

$$\text{where } \alpha \cong \frac{1}{2}\left[\frac{1}{\pi} ln\frac{R_{yy}}{R_{xx}} + \sqrt{(\frac{1}{\pi} ln\frac{R_{yy}}{R_{xx}})^2 + 4}\right].$$

Following the definition of the elastoresistance coefficient in the D_{6h} symmetry group with x along the [100] axis (see, Supplementary Note 4 for details), the elastoresistance coefficient along the E_{2g} symmetry channel ($m_{11} - m_{12}$) can be obtained by calculating

$$m_{11} - m_{12} = \frac{\left(\frac{\Delta\rho}{\rho_0}\right)_{xx} - \left(\frac{\Delta\rho}{\rho_0}\right)_{yy}}{\left(\varepsilon_{xx} - \varepsilon_{yy}\right)},$$

in the zero-strain limit of $(\varepsilon_{xx} - \varepsilon_{yy}) \to 0$. Here, the normalization factor $\rho_0 = \sqrt{\rho_{xx,0} \times \rho_{yy,0}}$ has been calculated by the zero-strain resistivity values $\rho_{xx,0}$ and $\rho_{yy,0}$ for each temperature, which were obtained from measuring the temperature dependence of a free-

standing crystal before attaching the sample to the piezo stack. Also, the zero-strain point of ε_{xx} and ε_{yy} has been obtained for each temperature by comparing ρ_{xx} and ρ_{yy} to the zero-strain resistivity values $\rho_{xx,0}$ and $\rho_{yy,0}$.

Supplementary Figure 7**c** shows the anisotropic change in normalized resistivity $N = \{(\Delta\rho_{xx}/\rho_0) - (\Delta\rho_{yy}/\rho_0)\}$ with response to anisotropic strain $(\varepsilon_{xx} - \varepsilon_{yy})$ at representative temperatures. It could be seen that a linear relationship holds near zero strain in the N vs. $(\varepsilon_{xx} - \varepsilon_{yy})$ data near the zero strain point. Supplementary Figure 7**d** shows the nematic susceptibility $\tilde{n}$ obtained from the modified Montgomery method (red unfilled diamonds), plotted alongside the data obtained from the four-probe method using two samples (green squares). It could be seen that both measurements reveal qualitatively similar results, indicating the reproducibility of the obtained data points. A comparison of the Curie-Weiss fitting parameters for the data in Supplementary Figure 7**d** is shown in Supplementary Table 3.

Supplementary Note 7. Determination of the 'zero strain point' in the four-probe measurement scheme

According to the definition of the elastoresistance coefficients[7], nematic susceptibility is defined by measuring the $\partial N/\partial(\varepsilon_{xx} - \varepsilon_{yy})$ value in the zero strain limit of $(\varepsilon_{xx} - \varepsilon_{yy}) \to 0$. However, in realistic conditions, differential thermal contraction of the piezo stack and the sample can in principle lead to a nonzero applied strain $(\varepsilon_{xx} - \varepsilon_{yy}) \neq 0$ even when zero voltage is applied to the stack. Therefore, it needs to be experimentally verified whether the 'actual zero strain point' for each measured temperature is within the dynamic measurement range of the piezo stack.

In order to determine the 'actual zero strain point' for each temperature, the temperature dependence of the zero strain resistance $R_0(T)$ has been experimentally obtained by measuring the resistance of a free-standing crystal, unstrained and unattached to any substrate. As this R_0 represents the resistance value of the 'actual zero strain point' for each

temperature, comparing the R_0 to the strained R values, namely R(V =150V) and R(V = -50V), would provide information of whether the 'zero strain point' is within the dynamic piezo stack range. Supplementary Figure 8**a** shows the temperature-dependence of the resistance values $R_{xx,0}$, R_{xx} (-50 V), and R_{xx} (150 V) of CsV_3Sb_5 plotted as red dashed lined, green circles, and blue triangles, respectively. Here, $R_{xx,0}$ corresponds to the R_0 value of the R_{xx} sample in the four-probe measurement scheme. Similarly, R_{xx}(-50 V) and R_{xx}(150 V) correspond to the strained R values of the same R_{xx} sample. Note that the $R_{xx,0}(T)$ data of the sample was taken before attaching the samples to the piezo stack. Although the three resistance curves are nearly identical to each other, a magnified figure in the inset of Supplementary Figure 8**a** indicates that the zero strain resistance $R_{xx,0}$ is indeed between R_{xx}(-50 V) and R_{xx}(150 V) within the temperature region of T = 240 K - 250 K.

In order to compare the three resistance values for each temperature, a R_{xx}(V)/ $R_{xx,0}$ curve is plotted in Supplementary Figure 8**b**. It could be seen that the zero strain resistivity as visualized in the red dashed line is between the strained resistivity values R_{xx}(-50 V) and R_{xx}(150 V) for the measured temperature range of 6K - 250K. This indicates that the zero strain point is within the dynamic range of the PZT stack for the 6K - 250K temperature range within study. Supplementary Figure 8**c**-**d** shows similar plots for the R_{yy} sample of the four-probe measurement scheme. For the R_{yy} configuration, the R_{yy}(-50 V) is larger than R_{yy}(150 V), as the application of positive voltage on the piezo stack shortens the sample along the current direction.

As the zero strain point is found within the dynamic range of the piezo stack, the next step is to check whether the nematic response is linear to the applied strain near this point. To investigate this feature, Supplementary Figure 3**b** of the main text is replotted in Supplementary Figure 9**a**-**e**, which shows the anisotropic resistivity response upon applied anisotropic strain for various temperatures. Here, we note that experimental values of $(\Delta R/R)$ has been obtained by $(\Delta R/R) = \{(R(V) - R(V=0))/R_0\}$, following the approach of H. –H. Kuo *et al.*[7]. It could be seen that the nematic response is linear to the applied strain in the entire

dynamic region of the piezo stack, which is shown by black solid lines indicative of a linear fit. Moreover, Supplementary Figure 9 further visualizes that the zero strain N value $N_0 = \{(R_{xx,0} - R_{xx}(V=0))/R_{xx,0} - (R_{yy,0} - R_{yy}(V=0))/R_{yy,0}\}$, indicated by the yellow stars is within the dynamic range between N(-50V) and N(150V) for the representative temperatures of 100 K, 94 K, 90 K, 60 K, and 36 K. These results confirm that the elastoresistance response of CsV_3Sb_5 is indeed linear back to zero strain in the four-probe measurement scheme.

Supplementary Note 8. Discussion of the proportionality constant α in the nematic susceptibility data

In a small strain limit, it has been shown that the nematic susceptibility $\tilde{n}$ becomes linearly proportional to the anisotropic change in the resistance $N \equiv (\Delta R/R)_{xx} - (\Delta R/R)_{yy}$ in response to anisotropic strain $(\varepsilon_{xx} - \varepsilon_{yy})$, which results in $\tilde{n} = \alpha \times \partial N / \partial(\varepsilon_{xx} - \varepsilon_{yy})$[7]. Here, α is a proportionality constant depending on microscopic details of the electronic structure. In general, α can be material dependent and also temperature dependent. However, in the case of CsV_3Sb_5, the perfect fitting of the m_{11}-m_{12} to the same functional form of $\tilde{n}$ predicted by the Ginzburg-Landau theory[14] (i. e. theoretical Curie-Weiss fit) down to the deviation temperature T_{nem} of ~36 K suggests that the constant α can be temperature independent within the experimental range of 36 K – 250 K. Moreover, the perfect Curie-Weiss fitting also holds for samples with $Cs(V_{1-x}Ti_x)_3Sb_5$ up to x = 0.03, suggesting that α can be regarded as a constant in CsV_3Sb_5 for a wide range of temperatures above ~T_{nem} and below 250 K for each doping and at least up to a few percent of disorder.

Supplementary Note 9. Sample dependence of T_c values for selected doping ratios

In order to check the sample dependency within the sample batches, the in-plane resistivity ρ_{ab} has been measured for three single crystals per each batch of x = 0, 0.0075, 0.02, and 0.05 in $Cs(V_{1-x}Ti_x)_3Sb_5$ (Supplementary Figure 10). It is noticed that the T_c values between

single crystals of each batch are rather reproducible; namely, the standard deviations of the measured T_c values are below 0.06 K for the four batches investigated. This supports that the variation of T_c among different samples in each batch is low enough to well reproduce the double-dome behavior.

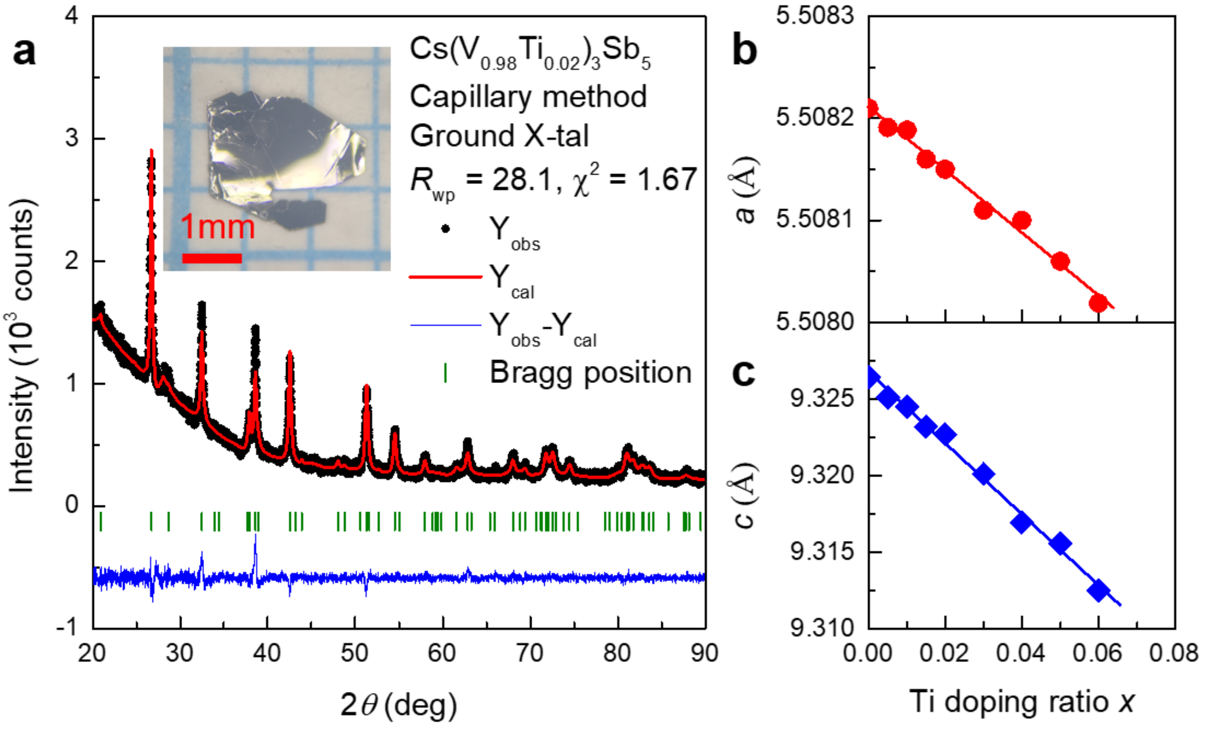

Supplementary Figure 1 | Capillary X-ray diffraction pattern of $Cs(V_{0.98}Ti_{0.02})_3Sb_5$ and the evolution of lattice parameters in $Cs(V_{1-x}Ti_x)_3Sb_5$. (**a**) Capillary x-ray diffraction patterns (black dots) and the corresponding Rietveld refinement result (red line) with R_{wp} = 28.1 and χ^2 = 1.67 of a $Cs(V_{0.98}Ti_{0.02})_3Sb_5$ single crystal. The subtracted patterns are shown as blue lines and the expected peak positions are shown as the green ticks. Inset shows a photograph of a $Cs(V_{0.98}Ti_{0.02})_3Sb_5$ single crystal placed on top of a graph paper with one unit of 1 mm (red scale bar). (**b**) and (**c**) show the evolution of *a* and *c* lattice parameters, respectively, with Ti doping ratio *x*.

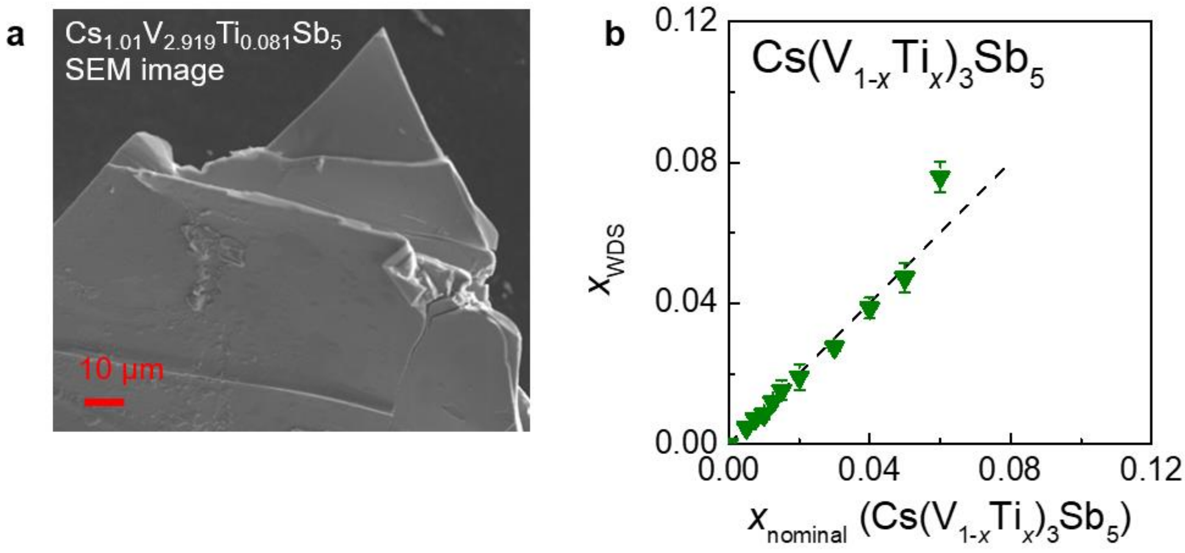

Supplementary Figure 2 | A scanning electron microscope image of $Cs_{1.01}(V_{2.919}Ti_{0.081})_3Sb_5$ and the evolution of the actual V: Ti doping ratios obtained from wavelength dispersive x-ray spectroscopy in $Cs(V_{1-x}Ti_x)_3Sb_5$. (**a**) Scanning electron microscope (SEM) image of a $Cs_{1.01}V_{2.919}Ti_{0.081}Sb_5$ single crystal. The relative ratio of Cs: V: Ti: Sb is obtained by a wavelength dispersive x-ray spectroscopy (WDS) analysis. The red horizontal bar indicates the scale, which is 10 μm in length. (**b**) A plot of nominal V: Ti ratio ($x_{nominal}$) vs. actual V: Ti ratio (x_{WDS}) as obtained from the WDS analysis. The error bar in green is obtained from the standard deviation of the V: Ti ratio obtained from 5 different measurement positions per each single crystal sample. The black dashed line refers to a linear guide line.

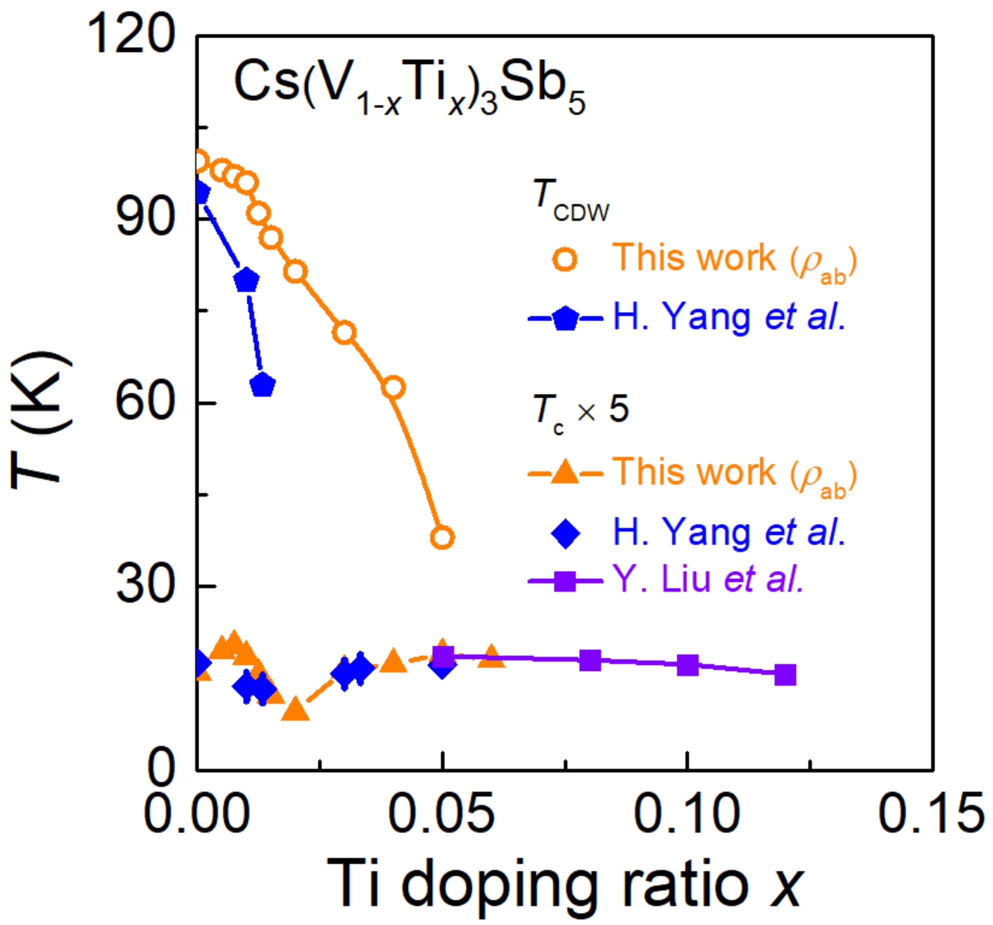

Supplementary Figure 3 | The phase diagrams of $Cs(V_{1-x}Ti_x)_3Sb_5$ obtained from various groups. The electronic phase diagram of $Cs(V_{1-x}Ti_x)_3Sb_5$ single crystals with Ti doping ratio x. The T_{CDW} and $T_c \times 5$ from this work are represented in orange circles and triangles, respectively. The blue octagons represent the T_{CDW} from ref. 5, while the blue diamonds and violet squares represent the $T_c \times 5$ from the refs. 5-6. The error bars in the blue diamonds indicate the variation of $T_c \times 5$ shown in ref. 5. For this work, the superconducting transition temperature T_c was determined by the criterion of $0.5\rho_N$ (ρ_N: normal-state resistivity). For the works from refs. 5-6, the superconducting transition temperature T_c was determined by the onset of the superconducting volume fraction χ.

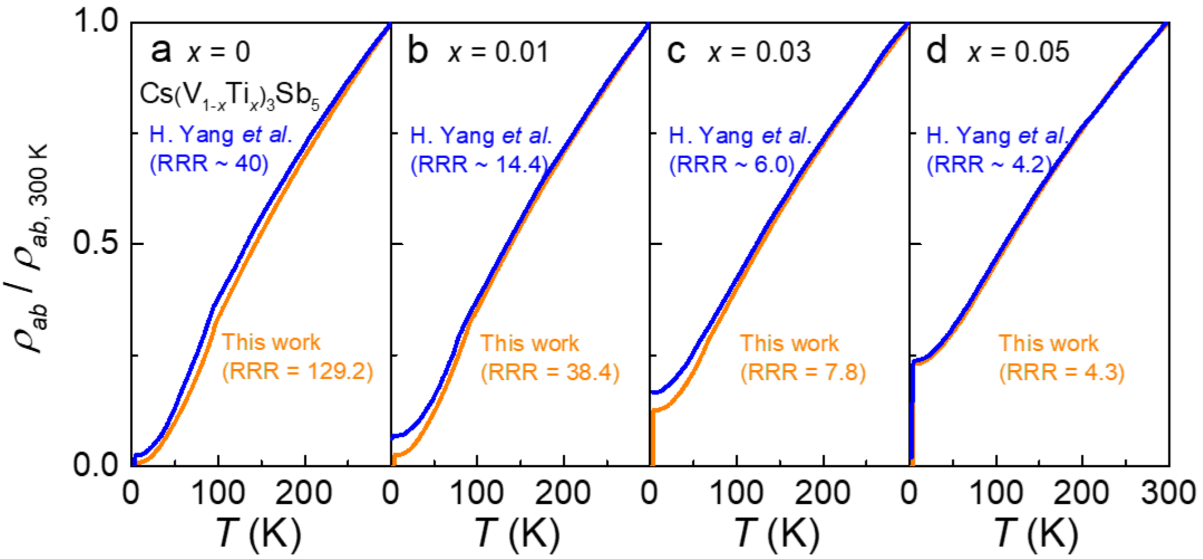

Supplementary Figure 4 | Comparison of the residual resistivity ratio RRR of $Cs(V_{1-x}Ti_x)_3Sb_5$ between this work and H. Yang *et al.* (**a**)–(**d**) Temperature dependence of the in-plane resistivity ρ_{ab} normalized to the value at 300 K (ρ_{ab}/ $\rho_{ab,300\ K}$) in $Cs(V_{1-x}Ti_x)_3Sb_5$ for $0 \leq x \leq 0.05$. The orange curve represents the data obtained by this work while the blue curve represents the data obtained from ref. 5. The residual resistivity ratio (RRR) for each sample are shown near the data.

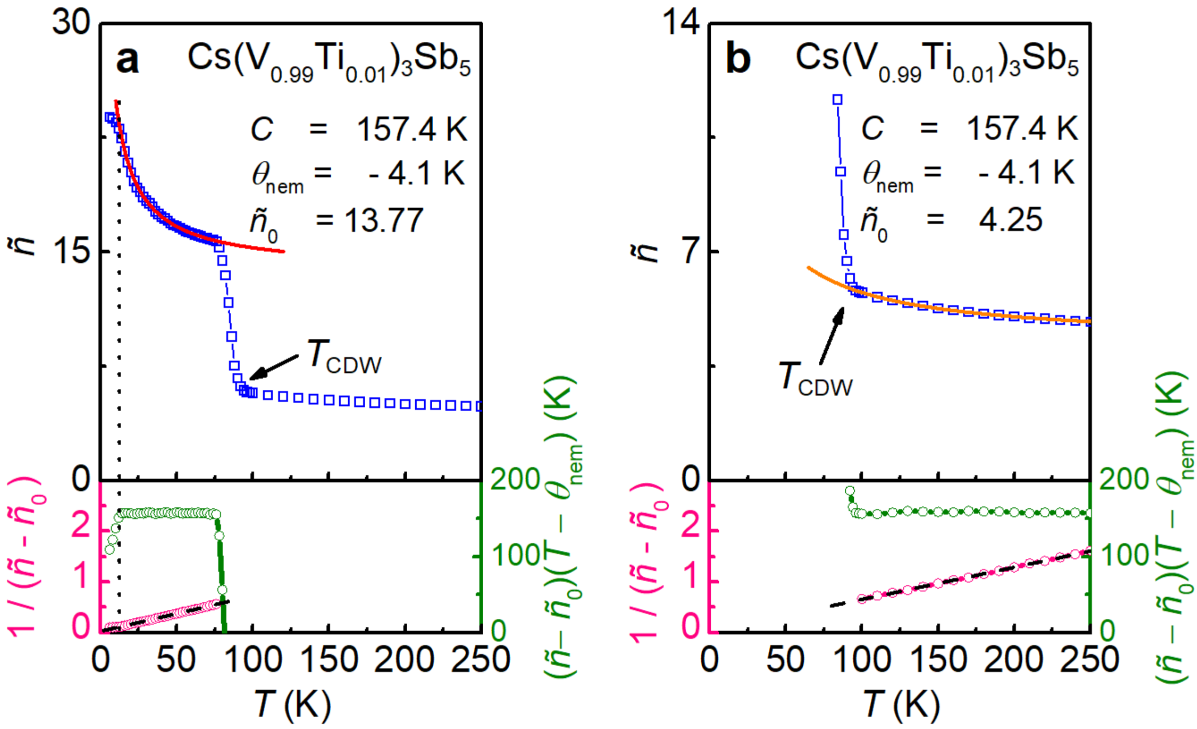

Supplementary Figure 5 | Temperature-dependent nematic susceptibility in $Cs(V_{0.99}Ti_{0.01})_3Sb_5$ below and above T_{CDW}. (**a**) (top panel) Temperature dependence of $\tilde{n}$ in $Cs(V_{0.99}Ti_{0.01})_3Sb_5$. A sharp jump in $\tilde{n}$ is observed near T_{CDW}. Below this jump, the data can be clearly fitted to the Curie–Weiss formula (Eq. (1) in the main text) with the best fitting parameters of C = 157.4 K, θ_{nem} = -4.1 K, and $\tilde{n}_0$ = 13.77 (red solid line). The black dotted line indicates the deviation temperature from a Curie–Weiss fit. (**b**) (top panel) A magnified figure of **a** visualizing the temperature dependence of $\tilde{n}$ above T_{CDW}. The data above T_{CDW} can be well described with the same fitting parameters of C = 157.4 K and θ_{nem} = -4.1 K, with a different choice of $\tilde{n}_0$ (orange solid line). (**a**) – (**b**) (bottom panel) Temperature dependence of $(\tilde{n} - \tilde{n}_0)^{-1}$ and $(\tilde{n} - \tilde{n}_0)(T - \theta_{nem})$ as represented by the pink and green open circles, respectively. The black dashed line refers to a linear guide line.

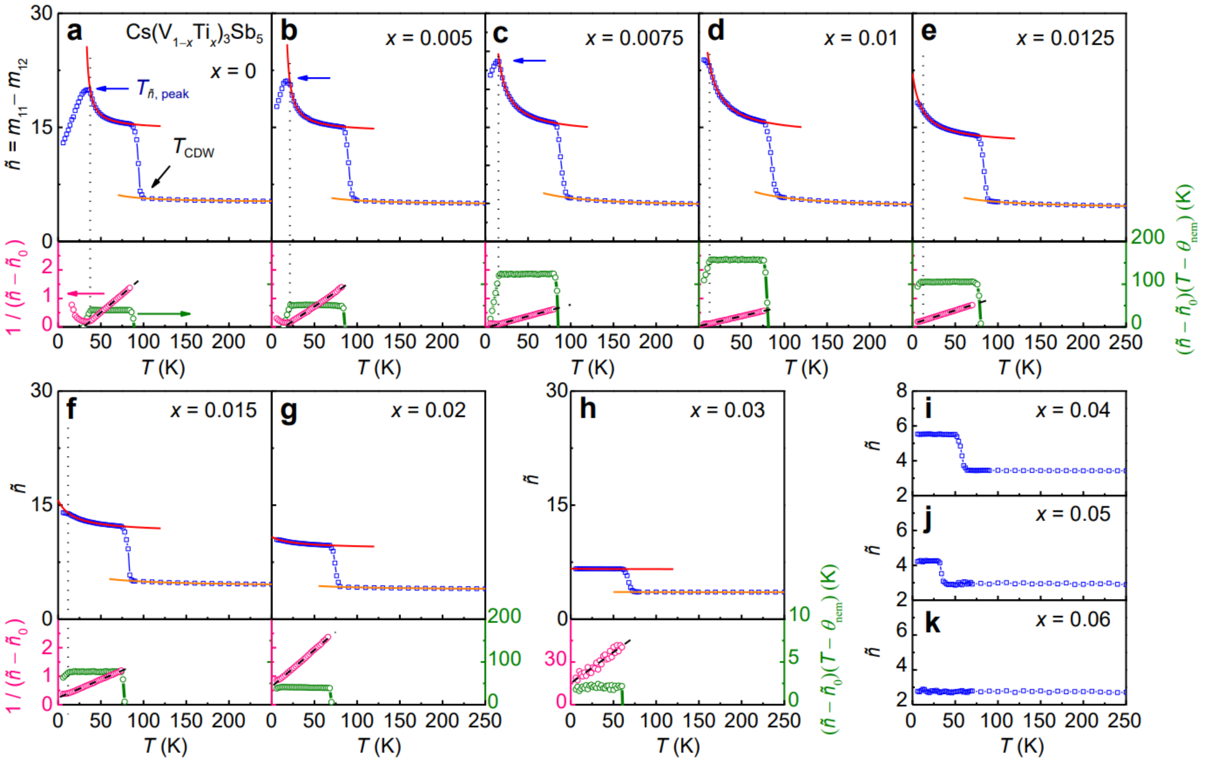

Supplementary Figure 6 | The temperature-dependent nematic susceptibility in $Cs(V_{1-x}Ti_x)_3Sb_5$ presented up to 250 K. (**a**)-(**h**) (top panel) Temperature dependence of $\tilde{n}$ in $Cs(V_{1-x}Ti_x)_3Sb_5$ for 0 ≤ x ≤ 0.03. A sharp jump in $\tilde{n}$ is observed near T_{CDW}. Below and above this jump, the data can be clearly fitted to the Curie–Weiss formula (Eq. (1) of the main text) (red and orange solid lines, respectively). The black dotted line indicates the deviation temperature from a Curie–Weiss fit, while the blue arrow indicates the peak temperature of $\tilde{n}$, $T_{\tilde{n},\ \mathrm{peak}}$. (**a**)-(**h**) (bottom panel) Temperature dependence of $(\tilde{n}-\tilde{n}_0)^{-1}$ and $(\tilde{n}-\tilde{n}_0)(T-\theta_{\mathrm{nem}})$ below T_{CDW}, represented by the pink and green open circles, respectively. The black dashed line refers to a linear guide line. (**i**)-(**k**) Temperature dependence of $\tilde{n}$ for 0.04 ≤ x ≤ 0.06.

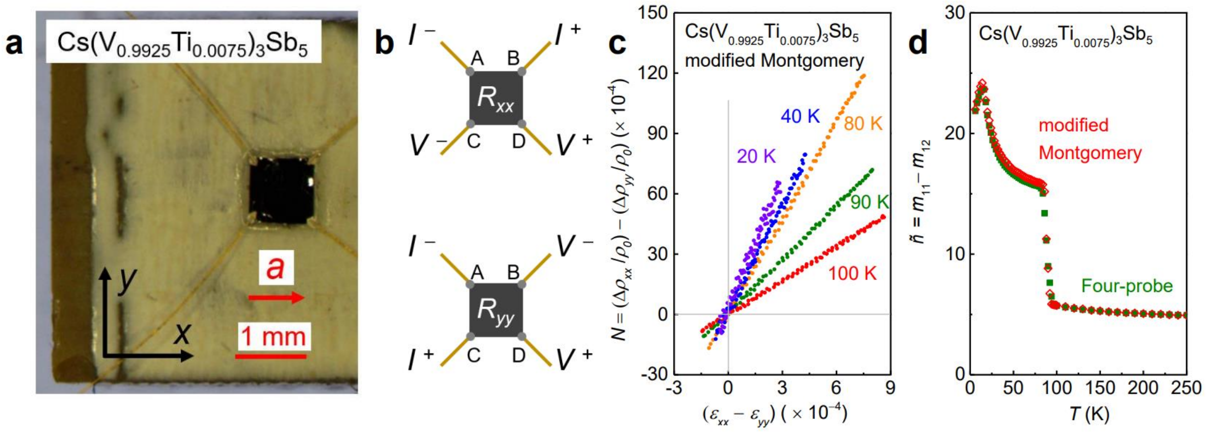

Supplementary Figure 7 | Experimental methods for the nematic susceptibility measurement using the modified Montgomery method and the temperature dependent nematic susceptibility in $Cs(V_{0.9925}Ti_{0.0075})_3Sb_5$. (**a**) A photo of the elastorestance setup using the modified Montgomery technique. A rectangular-shaped sample of $Cs(V_{0.9925}Ti_{0.0075})_3Sb_5$ with electric contacts on four corners are glued to the piezo stack with the *a*-axis along the Cartesian-*x* axis. A red scale bar indicates 1mm. (**b**) A schematic illustration of the measurement configurations for measuring R_{xx} and R_{yy}. (**c**) N vs. $(\varepsilon_{xx} - \varepsilon_{yy})$ plot of $Cs(V_{0.9925}Ti_{0.0075})_3Sb_5$ at several representative temperatures, measured by the modified Montgomery method. N exhibits a linear relationship to $(\varepsilon_{xx} - \varepsilon_{yy})$ near the zero strain point of $(\varepsilon_{xx} - \varepsilon_{yy}) \to 0$. (**d**) A comparison between the temperature dependent nematic susceptibility $\tilde{n}$ between the two different measurement configurations. The red diamonds (green squares) indicate the data obtained from the modified Montgomery (four-probe) method.

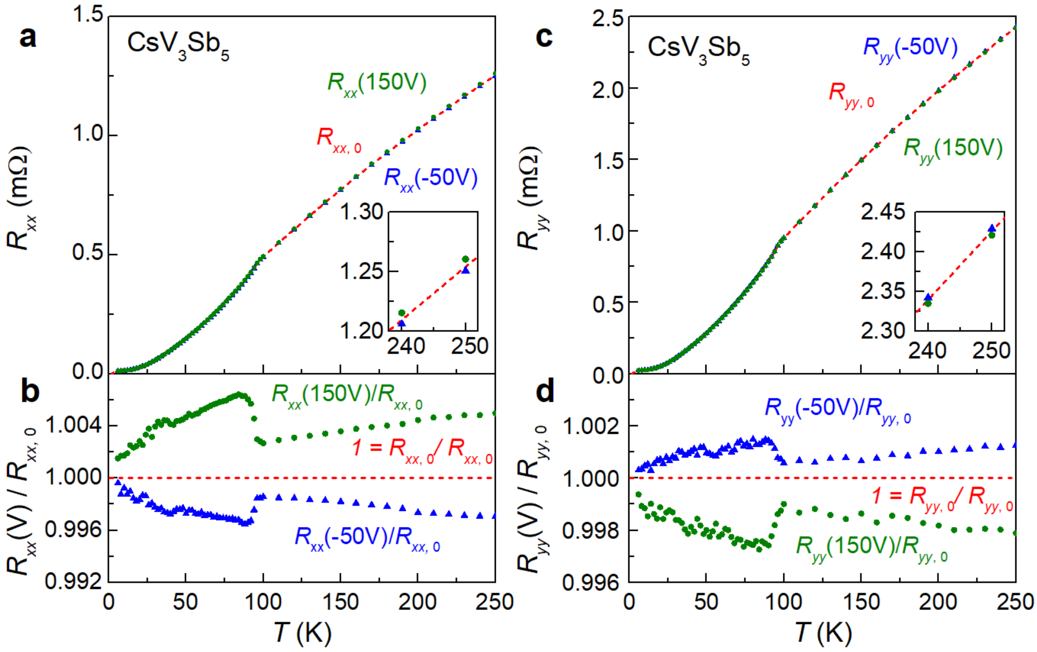

Supplementary Figure 8 | Temperature dependence of the zero strain resistance and the strained resistances of CsV_3Sb_5 single crystals. (**a**), (**c**) The temperature dependence of the zero strain resistance R_0 and the strained resistances R(-50 V), R(150 V) of CsV_3Sb_5 for two measurement configurations R_{xx} and R_{yy}, respectively. The red dashed lines, blue triangles and the green circles represent the R_0, R(-50 V), and R(150 V) values, respectively. The insets show the resistance values near 250 K. R(V)/R_0 curves for (**b**) the R_{xx} and (**d**) the R_{yy} measurement configurations.

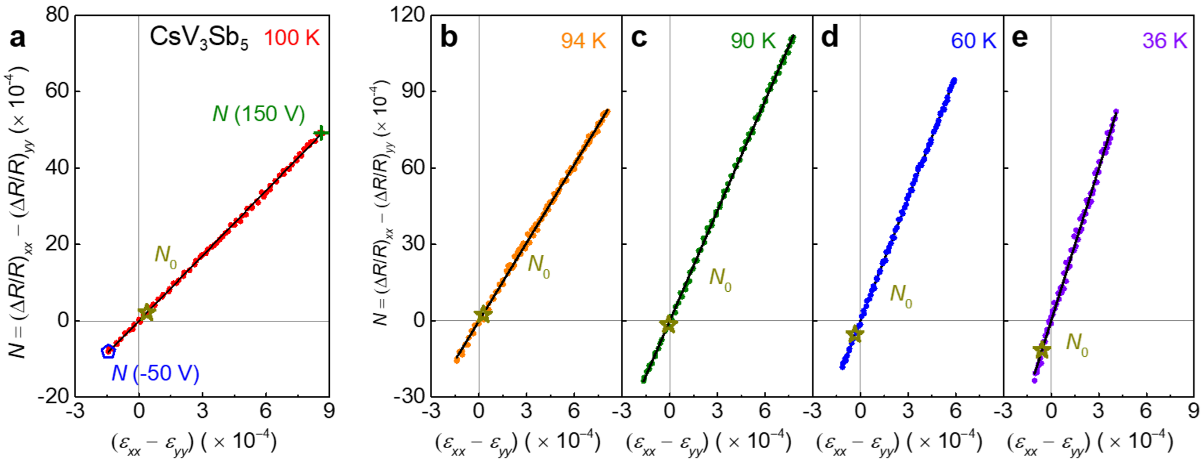

Supplementary Figure 9 | Anisotropic change of resistance *N* as a function of anisotropic strain $(\varepsilon_{xx} - \varepsilon_{yy})$ at several temperatures for CsV_3Sb_5. (**a**) *N* vs. $(\varepsilon_{xx} - \varepsilon_{yy})$ plot of CsV_3Sb_5 at 100 K. The black solid line shows the best fitting result for the linear dependence of $N = a \times (\varepsilon_{xx} - \varepsilon_{yy})$. The yellow star indicates the zero strain *N* value N_0, while the blue octagon and the green cross indicates the strained *N* values *N*(-50 V) and *N*(150 V), respectively. (**b**)-(**e**) *N* vs. $(\varepsilon_{xx} - \varepsilon_{yy})$ plot of CsV_3Sb_5 at 94 K, 90 K, 60 K, 36 K, respectively. The yellow star indicates the N_0 value for each temperature.

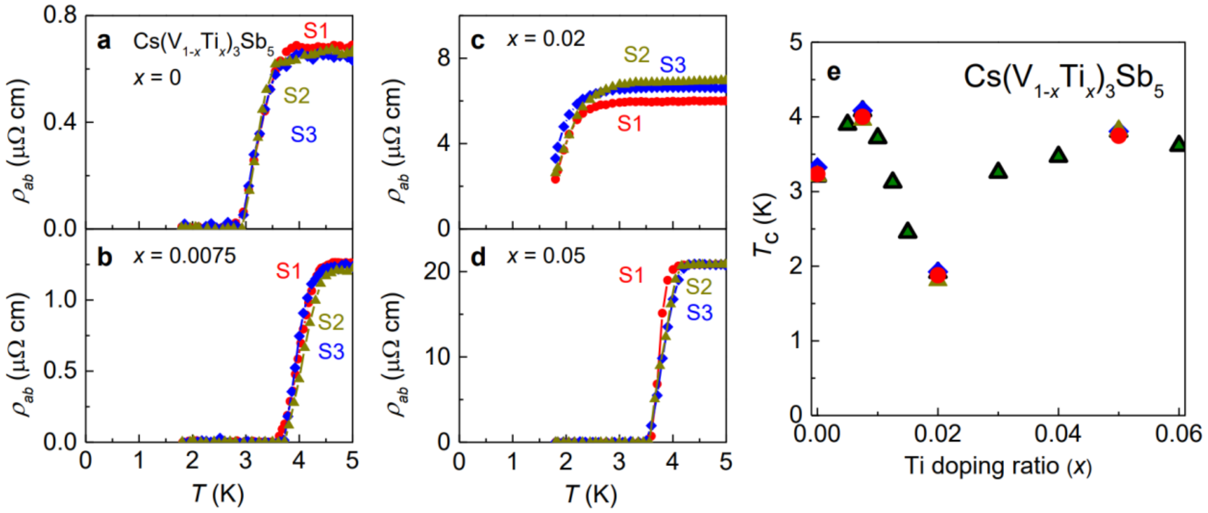

Supplementary Figure 10 | Sample dependence of the superconducting transition temperatures for selected batches in $Cs(V_{1-x}Ti_x)_3Sb_5$. Low-temperature behaviour of the in-plane resistivity ρ_{ab} in $Cs(V_{1-x}Ti_x)_3Sb_5$ of three samples in each batch for (**a**) x = 0, (**b**) x = 0.0075, (**c**) x = 0.02, (**d**) x = 0.05. Red circles, yellow triangles, and blue diamonds represent sample 1 (S1), sample 2 (S2), and sample 3 (S3), respectively. (**e**) Superconducting transition temperature T_c as a function of the Ti ratio x. Red circles, yellow triangles, and blue diamonds represent S1, S2, and S3, respectively. Green triangles represent T_c determined from Fig. 2 of the main text.

Supplementary Table 1 Comparison of the residual resistivity (ρ_0), residual resistivity ratio (RRR) and the charge density wave transition temperature (T_{CDW}) with values obtained from literature.

**reference**	**ρ_0 (μΩ cm)**	**RRR**	**T_{CDW} (K)**
this work	0.65	129.2	98
[2]	1	118	95.4
[3]	3.8	33	94
[4]	3.8	16	92.2

Supplementary Table 2 Summary of parameters obtained from analysis of $\tilde{n}$ in $Cs(V_{1-x}Ti_x)_3Sb_5$ for $0 \leq x \leq 0.03$. Here, T_{dev}, $T_{\tilde{n},\, peak}$, θ_{nem}, *C, and* $\tilde{n}_0$ indicates the deviation temperature, peak temperature, mean-field nematic transition temperature, Curie constant, and intrinsic anisotropy obtained from Curie-Weiss fits, respectively.

**Doping ratio *x***	T_{dev} **(K)**	$T_{\tilde{n},\, peak}$ **(K)**	θ_{nem} **(K)**	***C*** **(K)**	$\tilde{n}_0$	
					**~$T_{nem} < T <$ ~T_{CDW}**	**$T_{CDW} < T < 250$ K**
0	36	34	30.0	38.9	14.73	5.09
0.005	22	18	13.7	51.0	14.36	4.80
0.0075	18	14	3.6	123.7	13.98	4.43
0.01	12	-	-4.1	157.4	13.77	4.25
0.0125	8	-	-11.2	105.2	12.73	4.26
0.015	8	-	-18.2	77.3	11.39	4.29
0.02	-	-	-27.0	41.8	9.28	3.86
0.03	-	-	-41.0	2.1	6.58	3.56

Supplementary Table 3 Comparison of the fitting parameters between nematic susceptibility data obtained by the two different measurement techniques in $Cs(V_{0.9925}Ti_{0.0075})_3Sb_5$. Here, T_{dev}, $T_{\tilde{n},\ peak}$, θ_{nem}, *C, and* $\tilde{n}_0$ indicates the deviation temperature, peak temperature, mean-field nematic transition temperature, Curie constant, and intrinsic anisotropy obtained from Curie-Weiss fits, respectively.

	fitting parameters for $Cs(V_{0.9925}Ti_{0.0075})_3Sb_5$					
	T_{dev} (K)	$T_{\tilde{n},\ peak}$ (K)	θ_{nem} (K)	C (K)	$\tilde{n}_0$	
					$\sim T_{nem} < T < \sim T_{CDW}$	$T_{CDW} < T < 250$ K
modified Montgomery technique	18	14	3.4	125.2	14.30	4.43
Four-probe technique	18	14	3.6	123.7	13.98	4.43

Supplementary References

1. Ortiz, B. R. *et al*. New kagome prototype materials: discovery of KV_3Sb_5, RbV_3Sb_5, and CsV_3Sb_5. *Phys. Rev. Materials* **3,** 094407 (2019).

2. Zhang, W. *et al*. Emergence of large quantum oscillation frequencies in thin flakes of a kagome superconductor CsV_3Sb_5. *Phys. Rev. B* **106**, 195103 (2022).

3. Mi, X. *et al*. Multiband effects in thermoelectric and electrical transport properties of kagome superconductors AV_3Sb_5 (A = K, Rb, Cs). *New J. Phys.* **24,** 093021 (2022).

4. He, M. *et al*. Strong-coupling superconductivity in the kagome metal CsV_3Sb_5 revealed by soft point-contact spectroscopy. *Phys. Rev. B* **106,** 104510 (2022).

5. Yang, H. *et al*. Titanium doped kagome superconductor $CsV_{3-x}Ti_xSb_5$ and two distinct phases. Preprint at https://doi.org/10.48550/arXiv.2110.11228 (2021).

6. Liu, Y. *et al*. Doping evolution of superconductivity, charge order and band topology in hole-doped topological kagome superconductors $Cs(V_{1-x}Ti_x)_3Sb_5$. Preprint at https://doi.org/10.48550/arXiv.2110.12651 (2021).

7. Kuo, H. –H. Shapiro, M. C. Riggs, S. C. & Fisher, I. R. Measurement of the elastoresistivity coefficients of the underdoped iron arsenide $Ba(Fe_{0.975}Co_{0.025})_2As_2$. *Phys. Rev. B* **88,** 085113 (2013).

8. Nie, L. *et al*. Charge-density-wave-driven electronic nematicity in a kagome superconductor. *Nature* **604,** 59–64 (2022).

9. Hosoi, S. *et al*. Nematic quantum critical point without magnetism in $FeSe_{1-x}S_x$ superconductors. *Proc. Natl. Acad. Sci. U.S.A.* **113**, 29 (2016).

10. Gati, E. *et al*. Measurements of elastoresistance under pressure by combining *in-situ* tunable quasi-uniaxial stress with hydrostatic pressure. *Rev. Sci. Instrum.* **91**, 023904 (2020).

11. Xu, M. et al. Superconductivity and phase diagrams of $CaK(Fe_{1-x}Mn_x)_4As_4$ single crystals. *Phys. Rev. B* **105**, 214526 (2022).

12. Kuo, H. –H. Chu, J. –H. Palmstrom, J. C. Kivelson, S. A. & Fisher, I. R. Ubiquitous signatures of nematic quantum criticality in optimally doped Fe-based superconductors. *Science* **352,** 958-962 (2016).

13. dos Santos C. A. M. *et al*. Procedure for measuring electrical resistivity of anisotropic materials: A revision of the Montgomery method. *J. Appl. Phys.* **110**, 083703 (2011).

14. Chu, J. -H. Kuo, H. -H. Analytis, J. G. & Fisher, I. R. Divergent nematic susceptibility in an iron arsenide superconductor. *Science* **337,** 710–712 (2012).
